# Valid-NEO: A Multi-Omics Platform for Neoantigen Detection and Quantification from Limited Clinical Samples

**DOI:** 10.3390/cancers14051243

**Published:** 2022-02-28

**Authors:** Yuri Laguna Terai, Chun Huang, Baoli Wang, Xiaonan Kang, Jing Han, Jacqueline Douglass, Emily Han-Chung Hsiue, Ming Zhang, Raj Purohit, Taylor deSilva, Qing Wang

**Affiliations:** 1 Complete Omics Inc., Baltimore, MD 21227, USA; yuri@completeomics.com (Y.L.T.); rap1@umbc.edu (R.P.); tdesilv1@umbc.edu (T.d.); 2 Department of Lung Cancer, Tianjin Medical University Cancer Institute and Hospital, Tianjin 300060, China; yellowpure@126.com; 3 Institute of Endocrinology, Tianjin Medical University Chu Hsien-I Memorial Hospital and Tianjin Institute of Endocrinology, Tianjin 300134, China; blwang@tmu.edu.cn; 4 Renji Hospital, School of Medicine, Shanghai Jiao Tong University, Shanghai 200127, China; kangxiaonan@renji.com; 5 Tianjin Hospital of ITCWM Nankai Hospital, Tianjin 300100, China; hanjing6935@dingtalk.com; 6 Ludwig Center, Sidney Kimmel Comprehensive Cancer Center, The Johns Hopkins University School of Medicine, Baltimore, MD 21287, USA; jdouglass@jhmi.edu (J.D.); emily.hsiue@novartis.com (E.H.-C.H.); mzhang24@jhmi.edu (M.Z.)

**Keywords:** neoantigen, companion diagnostics, mass spectrometry, multi-omics assay, personalized cancer treatment

## Abstract

**Simple Summary:**

Neoantigens have emerged as highly personalized cancer therapeutic targets in recent years. Numerous studies have reported phenomenal therapeutic efficacy through treatments targeting cancer patient-specific neoantigens. Despite the growing interests, to identify druggable neoantigens is still largely dependent on genomic sequencing and AI algorithm-based prediction, which have been proven to be error-prone. Numerous reported mass spectrometry-based neoantigen assays utilized multi-gram levels of the tumor tissues, required invasive procedures, and were not practically feasible for most cancer patients, particularly patients that were non-operable or with early diseases, who may benefit most from immunotherapeutic approaches. Here we reported an integrated pipeline that can detect and quantify patient-specific neoantigens from a very limited amount of patient samples through a variety of innovative designs and optimizations. It may enable neoantigen-based therapeutics to benefit a broader spectrum of cancer and non-cancer patients.

**Abstract:**

The presentation of neoantigens on the cell membrane is the foundation for most cancer immunotherapies. Due to their extremely low abundance, analyzing neoantigens in clinical samples is technically difficult, hindering the development of neoantigen-based therapeutics for more general use in the treatment of diverse cancers worldwide. Here, we describe an integrated system, “Valid-NEO”, which reveals patient-specific cancer neoantigen therapeutic targets from minute amounts of clinical samples through direct observation, without computer-based prediction, in a sensitive, rapid, and reproducible manner. The overall four-hour procedure involves mass spectrometry analysis of neoantigens purified from tumor samples through recovery of HLA molecules with HLA antibodies. Valid-NEO could be applicable to the identification and quantification of presented neoantigens in cancer patients, particularly when only limited amounts of sample are available.

## 1. Introduction

Proteins encoded by genes carrying cancer-related mutations can be processed and mutation-bearing peptides can be presented on the cell surface in the context of human leukocyte antigen (HLA) molecules. Such peptides are called neoantigens. Neoantigens are truly cancer and patient specific, making them ideal anti-cancer therapeutic targets [1]. As the first class of neoantigen-targeting drugs, common neoantigens encoded by cancer driver hotspot mutations that are shared among a large number of patients have become highly popular therapeutic targets pursued by numerous pharmaceutical companies in the cancer immunotherapeutic field [2]. Neoantigens can be recognized by T cells via their T cell receptor, which is the foundation for cancer immunotherapies [1,2,3,4,5,6]. To identify potential targets to be exploited for immunotherapy, numerous techniques have been reported recently to reveal the repertoire of neoantigens, including genomic sequencing followed by algorithm-based predictions, deep profiling of the whole immunopeptidome through mass spectrometry or targeted detection approaches, as well as indirect assays on T cell reactivity [7,8,9]. We have previously reported a technology, Mutation Associated Neoantigen Selected Reaction Monitoring (MANA-SRM), as a basic research platform for neoantigen detection. MANA-SRM requires only approximately 200 million cultured tumor cells, which is over 10-fold more sensitive than previously published techniques available for this purpose [7,8,9]. It is an ideal tool for tissue culture-based neoantigen assays and has enabled the development of the first two off-the-shelf cancer vaccines targeting the most frequent mutation hotspots in the human tumor suppressor gene TP53 and the oncogene KRAS [10,11]. However, the sensitivity and robustness of MANA-SRM is still not feasible for routine clinical applications given the limited amount of tumor tissue available from biopsy or surgical resection. A more sensitive, rapid, and reproducible platform for neoantigen analysis from clinical samples is needed to more accurately and routinely implement neoantigen-based personalized cancer treatment.

**Figure 1:**
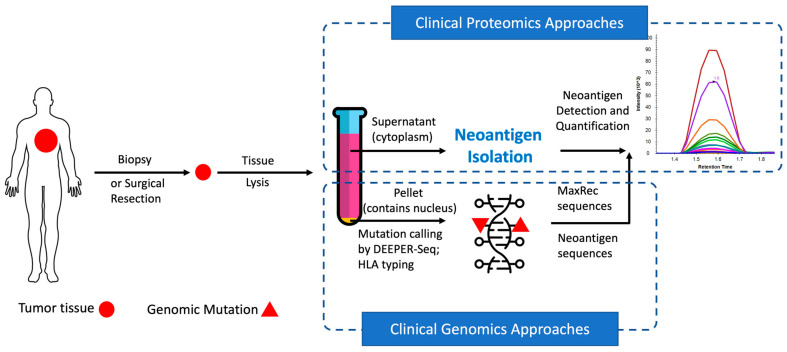
Valid-NEO multi-omics assay pipeline.

In recent years, mass spectrometry platforms have undergone a series of groundbreaking improvements [12,13,14]. Meanwhile, proteomic analysis is changing from a multi-hour assay to a short 21-min run with little compromise on coverage [15]. These improvements are dramatically revolutionizing mass spectrometry-based proteomics and making it more suitable for clinical applications. With further innovations in sample preparation and hardware re-configuration, we developed the Valid-NEO pipeline, an integrated multi-omics system for the detection and quantification of neoantigens from clinical samples without extensive manual sample processing (Figure 1). As an intact system, Valid-NEO requires little manual intervention, and therefore holds the potential to reduce operational variability from different diagnostic centers and hospitals in the development of personalized therapies for cancer patients.

## 2. Materials and Methods

### 2.1. Ethics Statement

This study was approved by the Institutional Review Boards for Human Research at Complete Omics Inc., Baltimore, MD, USA. (Baltimore, MD, USA) and BioIVT (Westbury, NY, USA), and complied with the Health Insurance Portability and Accountability Act.

### 2.2. Buffers Used for Neoantigen Isolation

Neoantigen Lysis (NL) buffer: RIPA buffer with the addition of 50 mM NaF, 80 uM β-glycerophosphate, 1 mM DTT, 1 mM PMSF, and 1 X Protease inhibitor mix (Roche); Neoantigen Capture (NC) buffer: modified RIPA buffer with 150 mM NaCl, 50 mM Tris (pH 7.4), 1% NP-40, 1 mM EDTA; Neoantigen Elution (NE) buffer: 10% Acetic Acid, 10 mM Ammonium Acetate; Neoantigen Neutralization (NN) buffer: 200 mM Tris-HCl (pH 8.5).

### 2.3. Tumor Sample Process and HLA Molecule Extraction

Human fresh frozen tumor samples (*n* = 9 patients) were obtained from BioIVT. Cancer types of the patients and patient-specific genetic mutation features of their tumors are listed in Supplementary Table S1. A chunk of frozen tissue (50 mg) was wrapped in at least four layers of aluminum foil and snap-frozen in liquid nitrogen. UniCeller (Complete Omics Inc., Baltimore, MD, USA), an in-house built device designed to apply strong impact to frozen tissue packs, was used to reduce the tissue chunk into a single-cell level powder. This procedure can be repeated five times. Neoantigen Lysis (NL) buffer (1 mL; Complete Omics Inc., Baltimore, MD, USA) was added to the frozen tissue powder and the tissue suspension was transferred to a protein lo-bind tube followed by five rounds of sonication with the Bioruptor 300 (energy level 4.5, duty step 30 s, and delay step 59 s). The tissue lysate was incubated on ice for 1 h, during which the suspension was pipetted up and down 20 times every 10 min, and one additional cycle of sonication was performed every 10 min. The tissue lysate was centrifuged at 4 °C for 30 min, and the clear supernatant was transferred to a new protein lo-bind tube. The supernatant containing the HLA molecules was diluted with 4 volumes of NC buffer (Complete Omics Inc., Baltimore, MD, USA) in preparation for HLA molecule isolation.

### 2.4. Construction of Valid-NEO

Valid-NEO is an integrated system composed of five steps essential for neoantigen detection, including (1) enrichment of HLA molecules; (2) elution of neoantigens from the antibody column; (3) rinsing/cleansing of neoantigens; (4) elution of neoantigens from the trap column; and (5) purification of neoantigens through an SEC column. This integrated system is composed of a tandem series of HPLC systems, one mass spectrometer, and a set of optimized buffers including the MaxRec system.

### 2.5. Online Enrichment of HLA Molecules through an Antibody-Column

Anti-HLA antibodies (clone W6/32) were immobilized on Protein A agarose beads (ThermoFisher Scientific, Waltham, MA) through a dimethyl pimelimidate (DMP)-based crosslinking reaction. Beads (50 mL) were then packed onto an HLA enrichment column and flushed with NC buffer (1 L; Complete Omics Inc., Baltimore, MD, USA). The HLA-neoantigen suspension was filtered through a 0.22 μm filter, diluted with 4 volumes of the NC buffer, and injected directly onto the HLA enrichment column. The flow-through was collected into a sample loop and re-injected onto the column. The injection was repeated four more times, for a total of five passes of the suspension through the antibody column. During the repeated loadings, HLA molecules were depleted from the mobile phase and captured by the column, while the HLA-suspension was gradually diluted with the NC buffer pumped into the system. The repeated loading ensured an efficient binding of the HLA molecules to the column, and the sequential dilution of the sample with the mobile phase facilitated an improved cleaning efficiency and reduced nonspecific binding. The antibody column was then flushed with NC buffer at 1 mL/min for 20 min to remove unbound proteins and impurities (including salts and detergents).

### 2.6. Online Elution of Neoantigen Peptides and Antibody Column Regeneration

Elution of the neoantigen peptides was performed with an increasing gradient (from 0 to 100% over a period of 5 min) of NE buffer (Complete Omics Inc., Baltimore, MD, USA) pumped through the column, followed by a continuous flush with 100% NE buffer at 1 mL/min for 2 min. The antibody column was then neutralized by running an increasing gradient (from 0 to 100 % over a period of 5 min) of NN buffer (Complete Omics Inc., Baltimore, MD, USA), followed by flushing with NC buffer for 1 h at 1 mL/min. The eluted HLA molecules and neoantigen peptides were then subjected to further purifications.

### 2.7. Online Isolation and Purification of Neoantigen Peptides

HLA eluate containing neoantigen peptides was injected to pass through a trap column for a total of five times, followed by washing with 10 mL of 0.1% formic acid. The cleaned peptides were eluted from the trap column through three cycles of acetonitrile gradients using mobile phase solvent A, 0.1% formic acid in water, and mobile phase solvent B, 0.1% formic acid in acetonitrile. The gradient started from 0% solvent B and increased to 60% solvent B over 30 s, and then decreased to 0% solvent B over 30 s. This one-min gradient step was repeated three times at the flow rate of 1 mL/min followed by a high-speed flush at 2 mL/min with 100% solvent B for 1 min. The flow-through was collected 30 s after the initial gradient change took place, and the collection was stopped 1 min after the flushing step ended. A total of 4.5 mL of neoantigen peptide suspension was collected with an estimated 30% acetonitrile and 0.1% formic acid. The collected neoantigen suspension was directly loaded onto an SEC column packed with 1.7 μm particles of 125 Å pore size (Waters, MA, USA). Before the analysis, the NEO-SEC ladder (Complete Omics Inc., Baltimore, MD, USA) was spiked into the system to define the boundaries for collecting the neoantigens. Signature chromatography peaks were monitored to indicate the starting point (a peak representing 2000 Da) and the ending point (a peak representing 800 Da) for the collection. Flow-through containing the isolated neoantigen peptides was collected and subject to lyophilization before mass spectrometry analysis.

### 2.8. Mass Spectrometry Method Development

Heavy isotope labeled neoantigen peptides flanking gene mutations in patient cancer genomes were synthesized. Optimization of the detection parameters was performed with a two-step approach. Step (1): All possible ions (first to last) of each peptide were detected with a theoretical collision energy as well as two additional collision energies at 5 eV below and above the theoretical value (three collision energy values in total for each transition). The highest abundance transitions were selected for the next round of optimization. Step (2): High abundance transitions selected from the previous step (>20 transitions for each charge status of the peptide target) were subject to a further optimization, in which, for each transition, nine collision energy values were tested, including the theoretical collision energy value as well as 4 steps of values below and above the theoretical value with a step-size of 2 eV. After two rounds of optimization, detection parameters were manually curated to avoid false positive signals from co-detected impurities in the Valid-NEO matrix prepared from a reference human tumor sample, and an average of 8 to 10 transitions were selected as signature transitions for each target. Before and after each batch of analysis, the Agilent 6495C Triple Quadrupole mass spectrometer was tuned using the manufacturer’s tuning mixture followed by the Complete360^®^ Tuning Booster (Complete Omics Inc., Baltimore, MD, USA). Before each assay, to ensure the stable and consistent performance of the mass spectrometer throughout the entire study, Complete360^®^ Performance Standard (Complete Omics Inc., Baltimore, MD, USA), a mixture of standard peptides at different concentrations across a wide range of masses (M/z 100–1400) and a broad range of hydrophobicities, was analyzed. A system performance score was documented before every run.

### 2.9. Pre-Conditioning the System to Ensure the Highest Sensitivity

In order to achieve the highest sensitivity for the assay, we developed a strategy to ensure minimal sample loss by pre-conditioning and passivating the system with peptides that are “similar” to the ones being detected. The peptides used to ensure the maximal recovery of the assay are called MaxRec peptides. A MaxRec prediction algorithm was created to generate MaxRec peptide sequences based on the sequences, hydrophobicity, and detectability (signal strengths detected in mass spectrometer) of the target peptides desired to be detected from the pipeline. MaxRec peptide sequences used in this study are shown (Supplementary Table S2). All MaxRec peptides were synthesized at a high purity (>99.9%). A buffer system containing MaxRec peptides at the concentration of 10 femtomole/μL was injected into the Valid-NEO pipeline before each assay. MaxRec peptides passed through the pipeline at much higher concentrations than would presumably be observed from the target peptides in clinical samples. Before clinical sample injection, the Valid-NEO pipeline was flushed with NC buffer for 30 min to deplete excess unbound MaxRec peptides.

### 2.10. Data Deposition

The data reported in this article have been deposited via ProteomeXchange in the PeptideAtlas SRM Experiment Library (PASSEL) (identifier PASS01588).

## 3. Results

The overall strategy is first to use HLA antibodies to collect HLA molecules potentially complexed with neoantigens and subsequently to purify the neoantigens from the complex for mass spectrometry analysis (Figure 1). To maximize the recovery of HLA molecules from tumor tissue samples, it is critical to rapidly homogenize the frozen tissue into a single-cell powder without thawing the sample. For this purpose, we devised an instrument, called the “UniCeller”, capable of pounding (~10,000 psi) frozen tissue chunks into a powder of single cells. Tissue powder samples produced with the UniCeller were quickly dissolved in Neoantigen Lysis (NL) buffer (Section 2), followed by repeated pipetting and programmed sonication (Section 2). Through this procedure, we were able to extract on average 98.38% of the HLA molecules from tissue samples, representing a greater recovery efficiency than when using traditional approaches, including the Dounce Homogenizer, the Probe Sonicator, and the Bead Ruptor (Supplementary Figure S1). The higher yield observed when using the UniCeller suggests that HLA molecules, as a protein complex predominantly located on the cell surface, may be vulnerable to temperature change and harsh mechanical force in liquid suspension during extraction. In addition, very few HLA molecules remained in the pellet from the UniCeller group (Supplementary Figure S1). This result indicates that a higher extraction efficiency can be achieved when a strong mechanical impact is applied to rapidly generate a single-cell level dry tissue powder, followed immediately by a moderate, but repeated, HLA extraction in lysis buffer.

The pellet obtained from the UniCeller tissue lysate was processed to extract genomic DNA (Figure 1). Single-stranded exomic regions of a selected panel of cancer driver genes were captured with our proprietary dual RNA probes and sequenced through the DEEPER-Seq pipeline as previously described [16]. A mutation call was made only when the point mutation was observed as a complementary pair of residues on both DNA strands derived from the same DNA duplex molecule and the procedure was conducted through our previously reported DEEPER-Seq next-generation sequencing (NGS) pipeline [16]. Nine tumor samples bearing hotspot mutations in highly frequently mutated cancer driver genes KRAS and TP53, as well as the slightly lower frequently mutated driver gene IDH2, were selected for further evaluation of potential neoantigen presentations.

**Figure 2:**
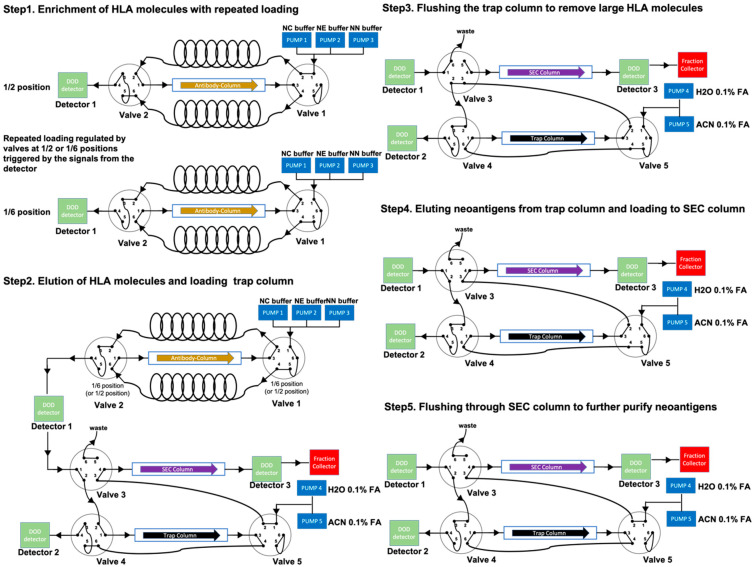
Scheme of neoantigen isolation and purification in the Valid-NEO pipeline.

Antibody column-based affinity chromatography is more efficient and cost-effective than conventional immunoprecipitation and was thus adopted in the Valid-NEO pipeline for enriching for HLA molecules [17]. To achieve a high enrichment efficiency, we packed an antibody-conjugated column with 50 mg of antibodies. In addition, repeated sample loadings were performed to ensure the binding between antibodies and HLA molecules in Neoantigen Capture (NC) buffer (Figure 2, Section 2). HLA complexes were eluted from the column in acidic Neoantigen Elution (NE) buffer (Section 2), and neoantigen peptides were dissociated from HLA molecules (Figure 2). The HLA enrichment column was then regenerated with Neoantigen Neutralization (NN) buffer. The column can be used for at least 27 assays with no significant loss in performance observed for the neoantigens analyzed in this study (Supplementary Figure S2). 

HLA molecules and other large proteins were separated from neoantigen peptides with a trap column packed with C18 small pore spherical silica particles (diameter 100Å). Neoantigens (average molecular weight ~1.5 kDa) are significantly smaller than HLA molecules (molecular weight ~41 kDa), and will therefore enter the pores and be efficiently retained by the C18 matrix predominately located within the pores. The majority of HLA molecules and other large proteins are not efficiently retained by the column. Neoantigens bound to the trap column were rinsed with 0.1% formic acid to remove HLA molecules and other impurities (Figure 2) and then eluted into a suspension composed of 30% acetonitrile and 0.1% formic acid. The suspension was spiked with the NEO-SEC ladder (Complete Omics Inc., Baltimore, MD, USA), containing two sets of peptides with signature molecular weights at 2000 and 800 Da (Section 2), and subjected to fractionation through a size exclusion column (SEC) (Figure 2). During elution, absorbance at the wavelength of 280 nm was constantly measured with a diode array detector (DAD). Collection of neoantigen fractions began after a signature peak at 2000 Da appeared and ended before an 800 Da signature peak appeared (Supplementary Figure S3). The flow-through between the two signature peaks was collected and lyophilized. The neoantigen sample was then subjected to Valid-NEO mass spectrometry analysis with pre-optimized conditions (Section 2).

To further improve the recovery ratio and the sensitivity of the pipeline, we invented the Maximum Recovery (MaxRec) System. The key element in MaxRec is a set of peptides which vary in sequence by only 1 or 2 amino acids from the target peptides and are resuspended in the MaxRec system at a much higher abundance than the endogenous neoantigens. MaxRec peptides are designed to mimic the physical characteristics of the target peptides, so as to saturate the potential for nonspecific binding in the system, thereby minimizing the loss of the target peptides caused by nonspecific interactions. Though they share similar physical characteristics with the target peptides, MaxRec peptides are chemically different, and can be easily distinguished from neoantigen targets based on the high-resolution mass analyzing capability of modern mass spectrometers (Figure 2). The Triple Quadrupole Mass Spectrometer in the Valid-NEO system is set up to be completely blind to MaxRec peptides, and the presence of the MaxRec system peptides will not reduce the sensitivity of the platform, which is a feature of this type of instrument.

We pre-conditioned the Valid-NEO neoantigen isolation system and spiked the samples with the MaxRec peptides (Supplementary Table S2). Using the MaxRec system, we significantly improved performance in the detection of all neoantigen peptides in this study, where the average increase in sensitivity was 27-fold (range from 2 to 67-fold) (Table 1).

It has been shown that almost all MHC class I associated neoantigens have a length of between 8 to 12 amino acids [18]. For each sample, all potential neoantigen sequences flanking the most prevalent mutation site on a cancer driver gene (a maximum of 50 possible neoantigen peptides for each missense mutation site) can be directly assayed for simultaneously without any prediction, thus preventing uncertainties (Supplementary Table S1). Using the Valid-NEO pipeline, we analyzed nine fresh frozen tumor samples to detect and quantify each patient’s personalized/unique neoantigens and compared the relative performance of MANA-SRM and our current integrated Valid-NEO pipeline (Table 1). The patients with KRAS mutations at the Q61 site had on average 6.6 copies of the neoantigen present on each tumor cell surface, and the neoantigens flanking the G12 site had an average of 32.1 copies present per tumor cell. The presentation of TP53 neoantigens was low, ranging from 1 to 8 copies per cell, which was similar to the presentation of IDH2 neoantigens at 6.1 copies of neoantigens per cell. Each assay was performed three times, and the reproducibility of the pipeline was thoroughly evaluated (Table 1, Supplementary Figure S2).

## 4. Discussion

Traditionally, cytotoxic chemotherapies have been the mainstay therapeutic agent for cancers, regardless of the individual genetic background of a given patient’s disease [19,20,21,22]. While cytotoxic chemotherapies are still the first line of treatment for many cancers, further molecular characterization of cancers has facilitated the development of small molecules or antibody-based agents that can treat a sub-population of the patients who share genetic features of their diseases [23,24,25]. With the development of NGS, it has become clear that each individual’s cancer has a unique genetic profile with varying degrees of overlap in cancer driver gene mutations among patients [26,27,28,29]. In recent years, highly personalized cancer therapeutic approaches, such as immunotherapy, have achieved success through targeting a patient’s specific neoantigens, offering hope with regard to the generalizability of such highly personalized treatments [4,6]. To reveal the neoantigen sequences needed for such personalized cancer therapeutics, algorithm-based or artificial intelligence (AI)-based predictions are often the choice when direct observation is impossible, but such predictions have proven to be unreliable for clinical applications [9,30]. Neoantigens can also be determined through co-culturing tumor cells with autologous T cells, followed by tetramer staining or peptide-pulsing assays. However, these functional assays are technically difficult and time consuming, and therefore cannot be readily adopted in clinical settings [8,31]. In Valid-NEO, no prediction is needed, and no cell culture is performed. In addition, while the neoantigens evaluated in this study are all presented in the context of class I major histocompatibility complexes (MHC I), a similar concept can be readily applied to class II MHC as previously described [9].

## 5. Conclusions

Valid-NEO is the only pipeline developed so far to directly validate and quantify neoantigens from a very limited input amount of clinical samples in a sensitive, rapid, and reproducible manner. This approach may help pave the way for the development of truly personalized cancer therapeutics that work for more patients with earlier diseases.

## Figures and Tables

**Table 1:** Neoantigen quantification from small quantities of fresh frozen tissue through different approaches.

Neoantigen	MANA-SRM Detected Ratio to Standards	Valid-NEO (w/o MaxRec) Detected Ratio to Standards	Valid-NEO (w/MaxRec)		
			Detected Ratio to Standards	Detected Abundance (Unit: Atto mole)	Copy Number per Tumor Cell (Assuming 50 M Cells)
KRAS_Q61H	non-detectable ± N/A	0.022 ± 0.006	1.045 ± 0.058	522.5	6.3
KRAS_Q61L	0.027 ± 0.002	0.053 ± 0.004	0.729 ± 0.037	364.5	4.4
KRAS_Q61R	non-detectable ± N/A	0.04 ± 0.004	1.495 ± 0.156	747.5	9.0
IDH2_R140Q	non-detectable ± N/A	0.015 ± 0.003	1.019 ± 0.057	509.5	6.1
TP53_Y220C	non-detectable ± N/A	0.054 ± 0.002	1.354 ± 0.014	677	8.2
TP53_R248W	0.011 ± 0.00006	0.086 ± 0.034	0.173 ± 0.031	86.5	1.0
TP53_R213L	non-detectable ± N/A	0.373 ± 0.057	0.662 ± 0.03	331	4.0
KRAS_G12V_9mer	non-detectable ± N/A	0.146 ± 0.022	5.458 ± 1.206	2729	32.9
KRAS_G12V_10mer	non-detectable ± N/A	0.141 ± 0.024	6.381 ± 1.693	3190.5	38.4
KRAS_G12D_9mer	0.0719 ± 0.012	0.145 ± 0.067	2.933 ± 1.227	1466.5	17.7
KRAS_G12D_10mer	0.113 ± 0.012	0.244 ± 0.104	6.518 ± 3.748	3259	39.3

## Data Availability

The data reported in this article have been deposited via ProteomeXchange in the Pep-tideAtlas SRM Experiment Library (PASSEL) (identifier PASS01588).

## Supplementary Materials

The following supporting information can be downloaded at: https://www.mdpi.com/article/10.3390/cancers14051243/s1, Figure S1: Comparison of lysis efficiency from different approaches; Figure S2: Valid-NEO reproducibility evaluation; Figure S3: HPLC Chromatogram for neoantigen purification through an SEC column.; Table S1: Neoantigens derived from mutations in cancer driver genes; Table S2: MaxRec peptides.
