# Supplementary material for: Valid-NEO: A Multi-Omics Platform for Neoantigen Detection and Quantification from Limited Clinical Samples
Source: Cancers (Basel). 2022 Feb 28;14(5):1243. doi: 10.3390/cancers14051243 (PMC8909145; doi:10.3390/cancers14051243)
Supplement: Supplementary file 1 [file cancers-14-01243-s001.zip › cancers-1596929-supplementary materials/cancers-1596929-supplementary materials.pdf]

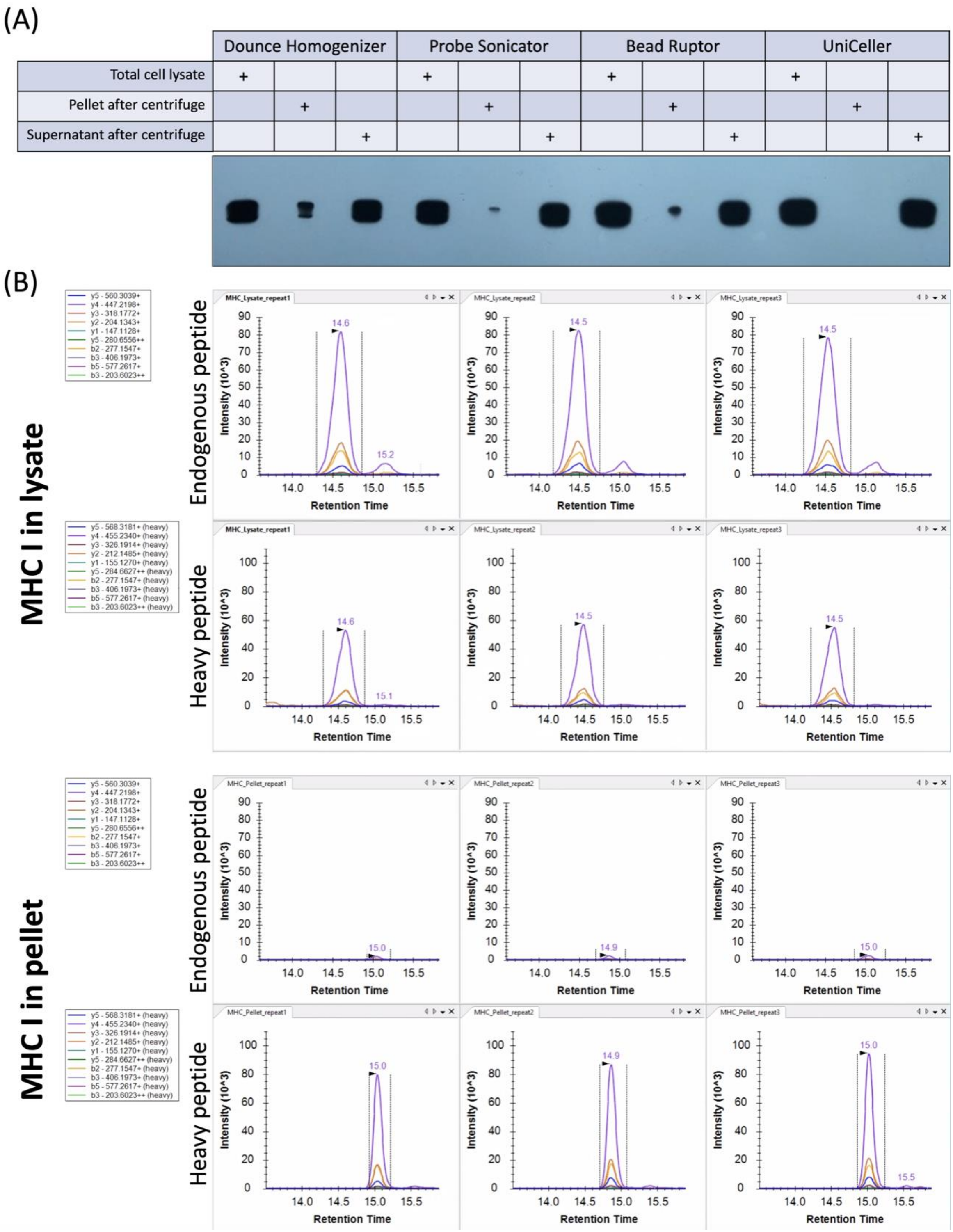

Figure S1. Comparison of lysis efficiency from different approaches. (**A**)Western blot analysis was performed to compare the efficiencies of HLA extractions using different approaches. The same amount (50 mg) of tumor tissue was processed through different approaches, including using Dounce Homogenizer, Probe Sonicator, Bead Ruptor, and UniCeller, for extracting HLA complexes. W6/32 antibody was used for the blot. (**B**) Targeted quantification of an MHC I conservative peptide (YLENGK) is performed using the total cell lysate and the pellet to quantify the extraction efficiency of the Valid-NEO lysis step.

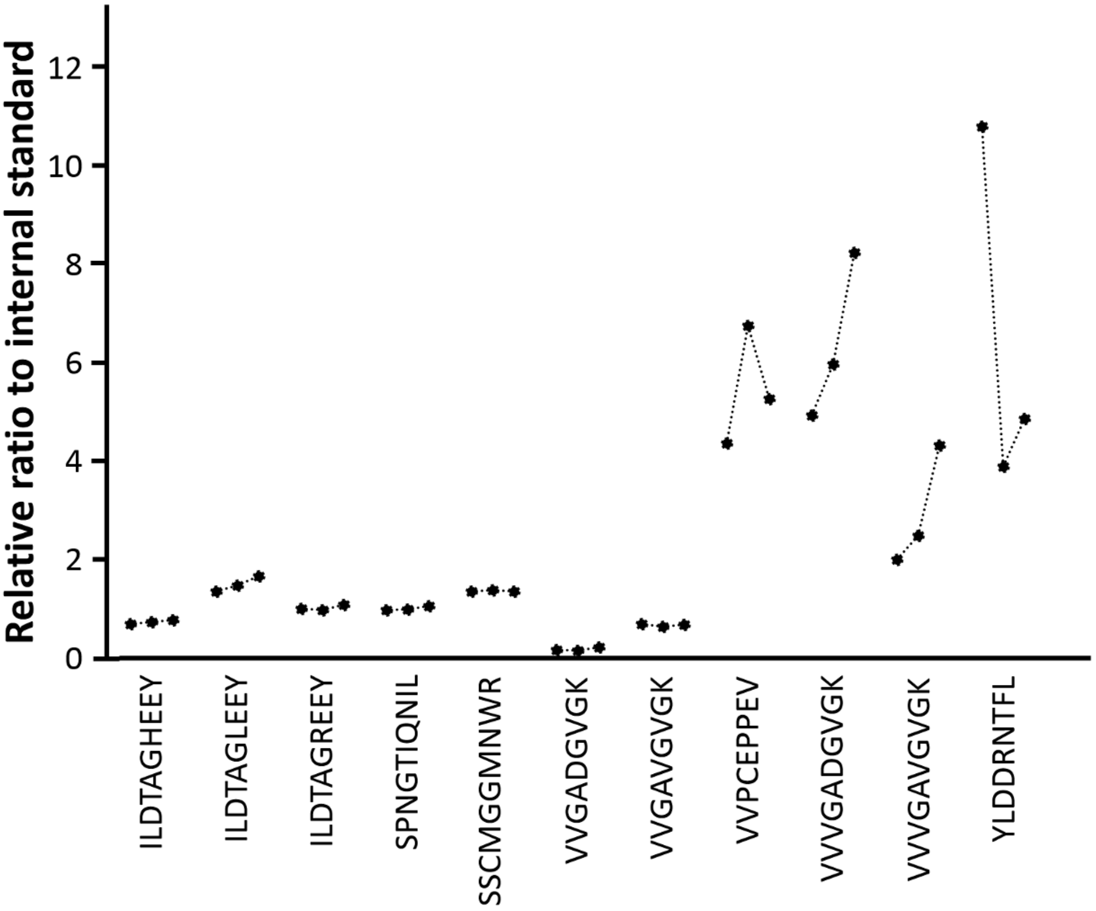

Figure S2. Valid-NEO reproducibility evaluation. Nine tumor samples were processed through the same Valid-NEO pipeline to evaluate their endogenous neoantigen presentations. All ten Valid-NEO assays were performed once, prior to the second replicate, and then again for the third replicate. Thus, between each given sample's replicates the other eight Valid-NEO assays were performed.

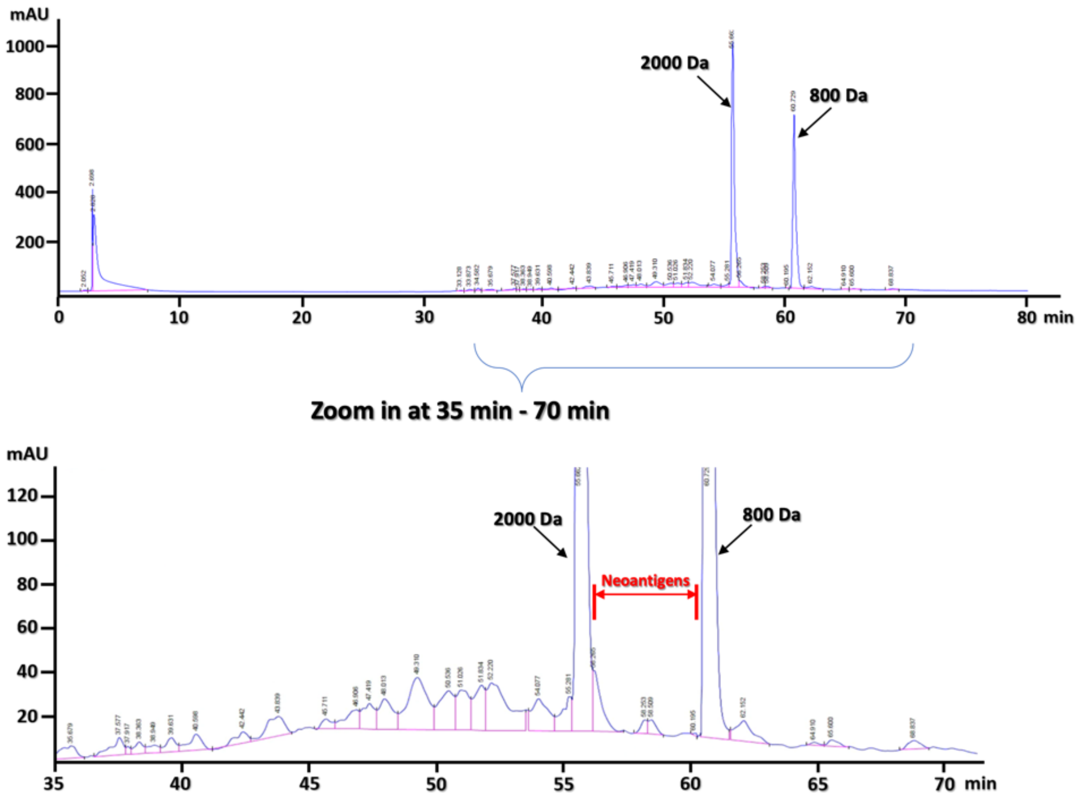

Figure S3. HPLC Chromatogram for neoantigen purification through an SEC column. Neoantigen samples were loaded to SEC column together with NEO-SEC ladders. Two signature peaks were observed at 2000 Da and 800 Da, which marked the boundaries for neoantigen-containing fractions.

Table S1. Neoantigens derived from mutations in cancer driver genes.

Sample ID	Cancer type	Cancer Driver Gene	Mutation	Mutation	12-AA neoantigen reference sequence	11-AA neoantigen reference sequence	10-AA neoantigen reference sequence	9-AA neoantigen reference sequence	8-AA neoantigen reference sequence
1	Human Large Intestine Adenocarcinoma Stage IA	*K-Ras*	**Q61H**	**HLA-A1**	TCLLDILDTAGHEEYSAMRDQYM	CLLDILDTAGHEEYSAMRDQY	LLDILDTAGHEEYSAMRDQ	LDILDTAGHEEYSAMRD	DILDTAGHEEYSAMR
					TCLLDILDTAGH	CLLDILDTAGH	LLDILDTAGH	LDILDTAGH	DILDTAGH
					CLLDILDTAGHE	LLDILDTAGHE	LDILDTAGHE	DILDTAGHE	ILDTAGHE
					LLDILDTAGHEE	LDILDTAGHEE	DILDTAGHEE	ILDTAGHEE	LDTAGHEE
					LDILDTAGHEEY	DILDTAGHEEY	ILDTAGHEEY	LDTAGHEEY	DTAGHEEY
					DILDTAGHEEYS	ILDTAGHEEYS	LDTAGHEEYS	DTAGHEEYS	TAGHEEYS
					ILDTAGHEEYSA	LDTAGHEEYSA	DTAGHEEYSA	TAGHEEYSA	AGHEEYSA
					LDTAGHEEYSAM	DTAGHEEYSAM	TAGHEEYSAM	AGHEEYSAM	GHEEYSAM
					DTAGHEEYSAMR	TAGHEEYSAMR	AGHEEYSAMR	GHEEYSAMR	HEEYSAMR
					TAGHEEYSAMRD	AGHEEYSAMRD	GHEEYSAMRD	HEEYSAMRD	
					AGHEEYSAMRDQ	GHEEYSAMRDQ	HEEYSAMRDQ		
					GHEEYSAMRDQY	HEEYSAMRDQY			
					HEEYSAMRDQYM				
2	Human Large Intestine Adenocarcinoma Stage IIB	*K-Ras*	**Q61L**	**HLA-A1**	TCLLDILDTAGLEEYSAMRDQYM	CLLDILDTAGLEEYSAMRDQY	LLDILDTAGLEEYSAMRDQ	LDILDTAGLEEYSAMRD	DILDTAGLEEYSAMR
					TCLLDILDTAGL	CLLDILDTAGL	LLDILDTAGL	LDILDTAGL	DILDTAGL
					CLLDILDTAGLE	LLDILDTAGLE	LDILDTAGLE	DILDTAGLE	ILDTAGLE
					LLDILDTAGLEE	LDILDTAGLEE	DILDTAGLEE	ILDTAGLEE	LDTAGLEE
					LDILDTAGLEEY	DILDTAGLEEY	ILDTAGLEEY	LDTAGLEEY	DTAGLEEY
					DILDTAGLEEYS	ILDTAGLEEYS	LDTAGLEEYS	DTAGLEEYS	TAGLEEYS
					ILDTAGLEEYSA	LDTAGLEEYSA	DTAGLEEYSA	TAGLEEYSA	AGLEEYSA
					LDTAGLEEYSAM	DTAGLEEYSAM	TAGLEEYSAM	AGLEEYSAM	GLEEYSAM
					DTAGLEEYSAMR	TAGLEEYSAMR	AGLEEYSAMR	GLEEYSAMR	LEEYSAMR
					TAGLEEYSAMRD	AGLEEYSAMRD	GLEEYSAMRD	LEEYSAMRD	
					AGLEEYSAMRDQ	GLEEYSAMRDQ	LEEYSAMRDQ		
					GLEEYSAMRDQY	LEEYSAMRDQY			
					LEEYSAMRDQYM				
3	Human Large Intestine Adenocarcinoma Stage IIB	*K-Ras*	**Q61R**	**HLA-A1**	TCLLDILDTAGREEYSAMRDQYM	CLLDILDTAGREEYSAMRDQY	LLDILDTAGREEYSAMRDQ	LDILDTAGREEYSAMRD	DILDTAGREEYSAMR
					TCLLDILDTAGR	CLLDILDTAGR	LLDILDTAGR	LDILDTAGR	DILDTAGR
					CLLDILDTAGRE	LLDILDTAGRE	LDILDTAGRE	DILDTAGRE	ILDTAGRE
					LLDILDTAGREE	LDILDTAGREE	DILDTAGREE	ILDTAGREE	LDTAGREE
					LDILDTAGREEY	DILDTAGREEY	ILDTAGREEY	LDTAGREEY	DTAGREEY
					DILDTAGREEYS	ILDTAGREEYS	LDTAGREEYS	DTAGREEYS	TAGREEYS
					ILDTAGREEYSA	LDTAGREEYSA	DTAGREEYSA	TAGREEYSA	AGREEYSA
					LDTAGREEYSAM	DTAGREEYSAM	TAGREEYSAM	AGREEYSAM	GREEYSAM
					DTAGREEYSAMR	TAGREEYSAMR	AGREEYSAMR	GREEYSAMR	REEYSAMR
					TAGREEYSAMRD	AGREEYSAMRD	GREEYSAMRD	REEYSAMRD	
					AGREEYSAMRDQ	GREEYSAMRDQ	REEYSAMRDQ		
					GREEYSAMRDQY	REEYSAMRDQY			
					REEYSAMRDQYM				
4	Human Lung Adenocarcinoma Stage IIB	*K-Ras*	**G12V**	**HLA-A3**	MTEYKLVVVGAVGVGKSALTIQL	TEYKLVVVGAVGVGKSALTIQ	EYKLVVVGAVGVGKSALTI	YKLVVVGAVGVGKSALT	KLVVVGAVGVGKSAL
					MTEYKLVVVGAV	TEYKLVVVGAV	EYKLVVVGAV	YKLVVVGAV	KLVVVGAV
					TEYKLVVVGAVG	EYKLVVVGAVG	YKLVVVGAVG	KLVVVGAVG	LVVVGAVG

					EYKLVVVGAVGV	YKLVVVGAVGV	KLVVVGAVGV	LVVVGAVGV	VVVGAVGV
					YKLVVVGAVGVG	KLVVVGAVGVG	LVVVGAVGVG	VVVGAVGVG	VVGAVGVG
					KLVVVGAVGVGK	LVVVGAVGVGK	VVVGAVGVGK	VVGAVGVGK	VGAVGVGK
					LVVVGAVGVGKS	VVVGAVGVGKS	VVGAVGVGKS	VGAVGVGKS	GAVGVGKS
					VVVGAVGVGKSA	VVGAVGVGKSA	VGAVGVGKSA	GAVGVGKSA	AVGVGKSA
					VVGAVGVGKSAL	VGAVGVGKSAL	GAVGVGKSAL	AVGVGKSAL	VGVGKSAL
					VGAVGVGKSALT	GAVGVGKSALT	AVGVGKSALT	VGVGKSALT	
					GAVGVGKSALTI	AVGVGKSALTI	VGVGKSALTI		
					AVGVGKSALTIQ	VGVGKSALTIQ			
					VGVGKSALTIQL				
5	Human Lung Adenocarcinoma Stage IA	*K-Ras*	**G12D**	**HLA-A3**	MTEYKLVVVGA**D**GVGKSALTIQL	TEYKLVVVGA**D**GVGKSALTIQ	EYKLVVVGA**D**GVGKSALTI	YKLVVVGA**D**GVGKSALT	KLVVVGA**D**GVGKSAL
					MTEYKLVVVGAD	TEYKLVVVGAD	EYKLVVVGAD	YKLVVVGAD	KLVVVGAD
					TEYKLVVVGADG	EYKLVVVGADG	YKLVVVGADG	KLVVVGADG	LVVVGADG
					EYKLVVVGADGV	YKLVVVGADGV	KLVVVGADGV	LVVVGADGV	VVVGADGV
					YKLVVVGADGVG	KLVVVGADGVG	LVVVGADGVG	VVVGADGVG	VVGADGVG
					KLVVVGADGVGK	LVVVGADGVGK	VVVGADGVGK	VVGADGVGK	VGADGVGK
					LVVVGADGVGKS	VVVGADGVGKS	VVGADGVGKS	VGADGVGKS	GADGVGKS
					VVVGADGVGKSA	VVGADGVGKSA	VGADGVGKSA	GADGVGKSA	ADGVGKSA
					VVGADGVGKSAL	VGADGVGKSAL	GADGVGKSAL	ADGVGKSAL	DGVGKSAL
					VGADGVGKSALT	GADGVGKSALT	ADGVGKSALT	DGVGKSALT	
					GADGVGKSALTI	ADGVGKSALTI	DGVGKSALTI		
					ADGVGKSALTIQ	DGVGKSALTIQ			
					DGVGKSALTIQL				
6	Human Head and Neck Squamous Cell Carcinoma Stage IIB	*TP53*	**Y220C**	**HLA-A2**	RNTFRHSVVVP**C**EPPEVGSDCTT	NTFRHSVVVP**C**EPPEVGSDCT	TFRHSVVVP**C**EPPEVGSDC	FRHSVVVP**C**EPPEVGSD	RHSVVVP**C**EPPEVGS
					RNTFRHSVVVPC	NTFRHSVVVPC	TFRHSVVVPC	FRHSVVVPC	RHSVVVPC
					NTFRHSVVVPCE	TFRHSVVVPCE	FRHSVVVPCE	RHSVVVPCE	HSVVVPCE
					TFRHSVVVPCEP	FRHSVVVPCEP	RHSVVVPCEP	HSVVVPCEP	SVVVPCEP
					FRHSVVVPCEPP	RHSVVVPCEPP	HSVVVPCEPP	SVVVPCEPP	VVVPCEPP
					RHSVVVPCEPPE	HSVVVPCEPPE	SVVVPCEPPE	VVVPCEPPE	VVPCEPPE
					HSVVVPCEPPEV	SVVVPCEPPEV	VVVPCEPPEV	VVPCEPPEV	VPCEPPEV
					SVVVPCEPPEVG	VVVPCEPPEVG	VVPCEPPEVG	VPCEPPEVG	PCEPPEVG
					VVVPCEPPEVGS	VVPCEPPEVGS	VPCEPPEVGS	PCEPPEVGS	CEPPEVGS
					VVPCEPPEVGSD	VPCEPPEVGSD	PCEPPEVGSD	CEPPEVGSD	
					VPCEPPEVGSDC	PCEPPEVGSDC	CEPPEVGSDC		
					PCEPPEVGSDCT	CEPPEVGSDCT			
					CEPPEVGSDCTT				
7	Human Large Intestine Adenocarcinoma Stage IIIA	*TP53*	**R248W**	**HLA-A11**	MCNSSCMGGMN**W**RPILTIITLED	CNSSCMGGMN**W**RPILTIITLE	NSSCMGGMN**W**RPILTIITL	SSCMGGMN**W**RPILTIIT	SCMGGMN**W**RPILTII
					MCNSSCMGGMNW	CNSSCMGGMNW	NSSCMGGMNW	SSCMGGMNW	SCMGGMNW
					CNSSCMGGMNWR	NSSCMGGMNWR	SSCMGGMNWR	SCMGGMNWR	CMGGMNWR
					NSSCMGGMNWRP	SSCMGGMNWRP	SCMGGMNWRP	CMGGMNWRP	MGGMNWRP
					SSCMGGMNWRPI	SCMGGMNWRPI	CMGGMNWRPI	MGGMNWRPI	GGMNWRPI
					SCMGGMNWRPIL	CMGGMNWRPIL	MGGMNWRPIL	GGMNWRPIL	GMNWRPIL
					CMGGMNWRPILT	MGGMNWRPILT	GGMNWRPILT	GMNWRPILT	MNWRPILT
					MGGMNWRPILTI	GGMNWRPILTI	GMNWRPILTI	MNWRPILTI	NWRPILTI
					GGMNWRPILTII	GMNWRPILTII	MNWRPILTII	NWRPILTII	WRPILTII
					GMNWRPILTIIT	MNWRPILTIIT	NWRPILTIIT	WRPILTIIT	
					MNWRPILTIITL	NWRPILTIITL	WRPILTIITL		

					NWRPILTIITLE	WRPILTIITLE			
					WRPILTIITLED				
8	Human Lung Adenocarcinoma Stage IIB	***TP53***	**R213L**	**HLA-A2**	RVEYLDDRNTFLHSVVVPYEPPE	VEYLDDRNTFLHSVVVPYEPP	EYLDDRNTFLHSVVVPYEP	YLDDRNTFLHSVVVPYE	LDDRNTFLHSVVVPY
					RVEYLDDRNTFL	VEYLDDRNTFL	EYLDDRNTFL	YLDDRNTFL	LDDRNTFL
					VEYLDDRNTFLH	EYLDDRNTFLH	YLDDRNTFLH	LDDRNTFLH	DDRNTFLH
					EYLDDRNTFLHS	YLDDRNTFLHS	LDDRNTFLHS	DDRNTFLHS	DRNTFLHS
					YLDDRNTFLHSV	LDDRNTFLHSV	DDRNTFLHSV	DRNTFLHSV	RNTFLHSV
					LDDRNTFLHSVV	DDRNTFLHSVV	DRNTFLHSVV	RNTFLHSVV	NTFLHSVV
					DDRNTFLHSVVV	DRNTFLHSVVV	RNTFLHSVVV	NTFLHSVVV	TFLHSVVV
					DRNTFLHSVVVP	RNTFLHSVVVP	NTFLHSVVVP	TFLHSVVVP	FLHSVVVP
					RNTFLHSVVVPY	NTFLHSVVVPY	TFLHSVVVPY	FLHSVVVPY	LHSVVVPY
					NTFLHSVVVPYE	TFLHSVVVPYE	FLHSVVVPYE	LHSVVVPYE	
					TFLHSVVVPYEP	FLHSVVVPYEP	LHSVVVPYEP		
					FLHSVVVPYEPP	LHSVVVPYEPP			
					LHSVVVPYEPPE				
9	Human Glioma Stage IIIA	***IDH2***	**R140Q**	**HLA-B7**	KKMWKSPNGTIQNILGGTVFREP	KMWKSPNGTIQNILGGTVFRE	MWKSPNGTIQNILGGTVFR	WKSPNGTIQNILGGTVF	KSPNGTIQNILGGTV
					KKMWKSPNGTIQ	KMWKSPNGTIQ	MWKSPNGTIQ	WKSPNGTIQ	KSPNGTIQ
					KMWKSPNGTIQN	MWKSPNGTIQN	WKSPNGTIQN	KSPNGTIQN	SPNGTIQN
					MWKSPNGTIQNI	WKSPNGTIQNI	KSPNGTIQNI	SPNGTIQNI	PNGTIQNI
					WKSPNGTIQNIL	KSPNGTIQNIL	SPNGTIQNIL	PNGTIQNIL	NGTIQNIL
					KSPNGTIQNILG	SPNGTIQNILG	PNGTIQNILG	NGTIQNILG	GTIQNILG
					SPNGTIQNILGG	PNGTIQNILGG	NGTIQNILGG	GTIQNILGG	TIQNILGG
					PNGTIQNILGGT	NGTIQNILGGT	GTIQNILGGT	TIQNILGGT	IQNILGGT
					NGTIQNILGGTV	GTIQNILGGTV	TIQNILGGTV	IQNILGGTV	QNILGGTV
					GTIQNILGGTVF	TIQNILGGTVF	IQNILGGTVF	QNILGGTVF	
					TIQNILGGTVFR	IQNILGGTVFR	QNILGGTVFR		
					IQNILGGTVFRE	QNILGGTVFRE			
					QNILGGTVFREP				

Table S2. MaxRec peptides.

ID	Neo-antigen peptide sequence	MaxRec peptides		
KRAS_Q61H	ILDTAGHEEY	ILDTAGHDEY	IVDTAGHEEY	ILDSAGHEEY
KRAS_Q61L	ILDTAGLEEY	ILDTAGLDEY	IVDTAGLEEY	ILDSAGLEEY
KRAS_Q61R	ILDTAGREEY	ILDTAGREDY	IVDTAGREEY	ILDSAGREEY
IDH2_R140Q	SPNGTIQNIL	SPNATIQNIL	SPNGTIQNIV	SPNGTVQNIL
TP53_Y220C	VVPCEPPEV	VVPCEPPDV	VVPCEPPEL	VIPCEPPEV
TP53_R248W	SSCMGGMNWR	SSCMAGMNWR	STCMGGMNWR	SSCMGGMQWR
TP53_R213L	YLDDRNTFL	YLEDRNTFL	YLDDRNTFV	YLDDRNSFL
KRAS_G12V_9mer	VVGAVGVGK	VVGAVGLGK	VVGAVAVGK	VVGGVGVGK
KRAS_G12V_10mer	VVVGAVGVGK	VVVGAVGLGK	VVVGAVAVGK	VVVGGVGVGK
KRAS_G12D_9mer	VVGADGVGK	VVGADGLGK	VVGADAVGK	VVGGDGVGK
KRAS_G12D_10mer	VVVGADGVGK	VVVGADGLGK	VVVGADAVGK	VVVGGDGVGK
