# Supplementary figures and images for: Valid-NEO: A Multi-Omics Platform for Neoantigen Detection and Quantification from Limited Clinical Samples
Source: Cancers (Basel). 2022 Feb 28;14(5):1243. doi: 10.3390/cancers14051243 (PMC8909145; doi:10.3390/cancers14051243)

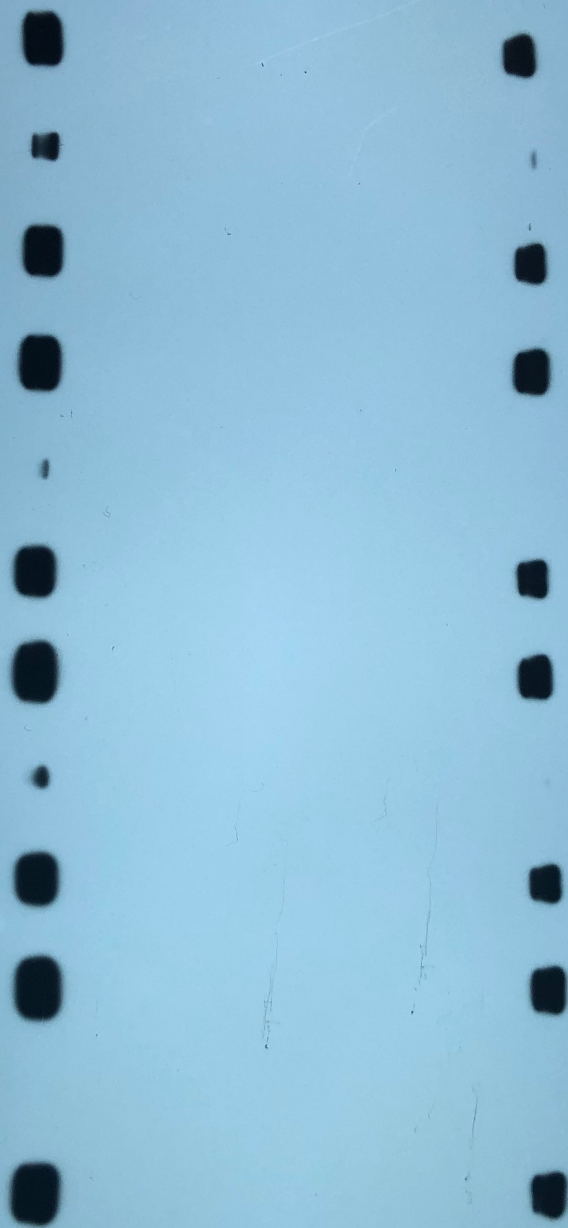

Supplement: Supplementary file 1 [file cancers-14-01243-s001.zip › cancers-1596929-supplementary materials/cancers-1596929-original-images.pdf]
